# Long‐term effects of air pollution on hospital admissions and mortality for chronic obstructive pulmonary disease in Beijing, China

**DOI:** 10.1111/crj.13656

**Published:** 2023-07-01

**Authors:** Rui‐xia Zhu, Jin Chen

**Affiliations:** ^1^ Respiratory Department, Fuxing Hospital Capital Medical University Beijing China

**Keywords:** air pollution, COPD hospital admissions, COPD mortality

## Abstract

**Objective:**

We aimed to clarify the association between air pollution and hospital admissions for chronic obstructive pulmonary disease (COPD) and mortality in Beijing, China.

**Methods:**

In this retrospective study, we recruited 510 COPD patients from 1 January 2006 to 31 December 2009. The patient data were obtained from the electronic medical records of Peking University Third Hospital in Beijing. Air pollution and meteorological data were obtained from the Institute of Atmospheric Physics of the Chinese Academy of Sciences. Monthly COPD hospital admissions, mortality and air pollution data were analysed using Poisson regression in generalised additive models adjusted for mean temperature, pressure and relative humidity.

**Results:**

There were positive correlations between sulfur dioxide (SO_2_), particulate matter with an aerodynamic diameter ≤ 10 μm (PM_10_) and COPD hospital admissions in the single‐pollutant model. An increase of 10 μg/m^3^ in SO_2_ and PM_10_ were associated with an increase of 4.053% (95% CI: 1.470–5.179%) and 1.401% (95%CI: 0.6656–1.850%) in COPD hospital admissions. In the multiple‐pollutant model [SO_2_ and nitrogen dioxide (NO_2_) combinations], there was only a positive correlation between SO_2_ and COPD hospital admissions. An increase of 10 μg/m^3^ in SO_2_ were associated with an increase of 1.916% (95% CI: 1.118–4.286%) in COPD hospital admissions. There was no correlation between three pollutant combinations and COPD hospital admissions.

We did not find correlations between air pollution and COPD mortality in either single‐ or multiple‐pollutant models.

**Conclusions:**

SO_2_ and PM_10_ may be important factors for the increase in COPD hospital admissions in Beijing, China.

## Graphical abstract

We conducted air pollutants (SO_2_, NO_2_ and PM_10_) and COPD hospitalizations and mortality in Beijing, China. SO_2_ and PM_10_ may be important factors for the increase in COPD hospital admissions. In recent years, air pollution has decrease significantly in Beijing.

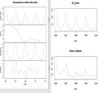

## 1 INTRODUCTION

Chronic obstructive pulmonary disease (COPD) is one of the most common chronic respiratory disease and a major health problem in the developed and developing countries. According to the China Pulmonary Health (CPH) study, the prevalence of COPD in Chinese people aged ≥20 years was 8.6%, and aged ≥40 years old is 13.7%.^1^ COPD affects nearly 100 million adults and is the third leading cause of death in China.^2^ Tobacco smoking is by far the most common factor in the etiology of COPD. However, additional causal associations have also been found among both smokers and nonsmokers, including dust, gas, vapor, and biological and chemical exposures.^3^, ^4^ Of all the other risk factors, particulate air pollution is now recognised as a potential environmental risk factor that causes many health hazards for respiratory diseases, especially COPD.

Numerous epidemiological studies have demonstrated that short‐ and long‐term exposure to air pollution is associated with COPD hospital admissions, acute exacerbation and mortality.^5^, ^6^, ^7^ Dong et al.^8^ demonstrated that a 10‐μg/m^3^ increase in PM_10_, SO_2_ and NO_2_ was associated with a 0.25% (95%CI: 0.01–0.49%), 1.67% (95%CI: 0.54–3.93%) and 1.37% (95%CI: 0.25–2.51%) increase in COPD outpatient visits, respectively. Zhang et al.^9^ found that every 10‐μg/m^3^ increase in pollutants, the majority of the summary estimates for COPD hospital admissions were in the order of (ozone 3) O_3_ > (particulate matter with aerodynamic diameter ≤ 2.5 um) PM_2.5_ > NO_2_ > PM_10_ > SO_2_. However, inconsistent and different results have been found for air pollution and COPD hospital admissions and mortality in different countries and even within the same regions. In 2012, our team conducted a meta‐analysis of PM_10_ and COPD hospitalisations and mortality. We found that a 10‐μg/m^3^ increase in PM_10_ was associated with a 2.7% (95%CI: 1.9–3.6%) increase in COPD hospitalisations and a 1.1% (95%CI: 0.8–1.4%) increase in COPD mortality.^10^ In 2020, we conducted another meta‐analysis of PM_2.5_ and COPD hospitalisations and mortality. We found that a 10‐μg/m^3^ increase in PM_2.5_ was associated with a 2.5% (95%CI:1.8–3.2%) increase in COPD hospitalisations and a 1.5% (95%CI: 0.9–2.2%) increase in COPD mortality.^11^

However, most of the aforementioned investigations have been performed in America and Europe, where air pollutant levels are much lower than those in developing countries. Few studies conducted in China have explored the relationship between air pollution and COPD hospital admissions and mortality. Rapid development in Beijing has resulted in a significant increase in air pollution. Therefore, it is important to determine the effects of air pollution on COPD. Beijing, the capital of China, has a population of over 11 million and is located at 39°56′N′ and 116°20′E′. The Haidian District is located in the northwest of Beijing, which belongs to the city center, and there are many tall buildings that can block air flow. Haidian District is socioeconomically highly developed and has a heavy and complex flow of traffic, as well as a large floating population. Air pollution levels are very high in the Haidian District. Most residents of Haidian District depend on the Peking University Third Hospital as a health resource. Data on COPD hospital admissions and mortality at this hospital can thus reflect the general health condition of the Beijing population. The first author was a graduate student at Peking University Third Hospital in Haidian District from 2009 to 2012, and COPD data could therefore be obtained during the study period.

In this study, we assessed the effects of air pollution on hospital admissions and mortality among patients with COPD at Peking University Third Hospital, from 1 January 2006 to 31 December 2009 in Beijing, China. We assessed the patterns of exposure–response relationships for three pollutants (monthly SO_2_, NO_2_ and PM_10_) and COPD hospital admissions and mortality in single and multiple‐pollutant models.

## 2 METHODS

### 2.1 Data of COPD collection

The data of patients with COPD between January 2006 and December 2009 were obtained from the Medical Record Room, Peking University Third Hospital, an affiliate of the Peking University Health Science Center. The collected data included sex, age, address, telephone number, education, smoking history, height, weight, complications, lung function [including forced expiratory volume in 1 s (FEV_1_)]; forced vital capacity (FVC), [FEV_1_%predicted (FEV_1_%pred)]; and date of death. The effects of particulate air pollution may have lagged; we followed the health condition of patients with COPD until 31 May 2010. The original purpose of this study was to compare the relationship between air pollution and COPD hospitalisations and mortality between 2006–2009 and 2016–2019.

The inclusion criteria were (1) patients with a diagnosis of COPD according to the American Thoracic Society (ATS)^12^ and Global Initiative for Obstructive Lung Disease (GOLD)^13^ or according to the International Classification of Diseases, 10th Revision^14^; (2) living in the district for 4 years and had been away for less than 6 months during the study period; (3) patients with complete basic information, such as residential address and phone numbers; and (4) all the participants that lived more than 500 m away from the main road without exposure to fuels or occupational exposure. The residence and surrounding environment of all participants were confirmed via phone.

The exclusion criteria were as follows: (1) unclear diagnosis of COPD; (2) presence of complications, such as malignant tumour, asthma, tuberculosis (TB), severe cardiovascular and cerebrovascular diseases, hepatic and renal insufficiency; and (3) missing or insufficient basic information.

As this was a retrospective study, we waived the requirement for ethical approval and informed patient consent from the Ethics Committee of Peking University Third Hospital.

### 2.2 Air pollution and meteorological data collection

Monthly records of PM_10_, SO_2_ and NO_2_ in the Haidian District in Beijing from 1 January 2006 to 31 December 2009 were obtained from the Institute of Atmospheric Physics, Chinese Academy of Science. Meteorological data, including the daily mean temperature, pressure and relative humidity were obtained from the Institute of Atmospheric Physics, Chinese Academy of Sciences.

### 2.3 Statistical analysis

To estimate the effects of air pollution on COPD hospital admissions and mortality, we used Poisson regression in generalised additive models (GAM) with R version 3.6.0 software for Windows (www.r-project.org). The models were adjusted for the daily mean temperature, pressure and relative humidity. The choice of degrees of freedom (df) for the smoothing spline of any weather variable was between 3 df. Before the analysis, we first fitted the original data using log_10_‐trasformed to improve normality and stabilise variance. We fitted nonparametric smoothing terms (using the smoothing spine function) for daily mean temperature, pressure and relative humidity. After controlling for the weather variables, we introduced each pollutant separately into the single‐pollutant model. We also fitted models with different combinations of pollutants (two and three pollutants per model) to assess the stability of the effect, which was analysed in the multiple‐pollutant model.

The monthly number of patients hospitalised for COPD relative to the general population was a small probability event with an approximate Poisson distribution. The statistics approximate at Poisson distribution. We used the model described below to explore the relationship between air pollutants and COPD hospital admissions and mortality.

$$\log\left[E\;\left(\mathrm{Yt}\right)\right]=\beta \;\left(X_{t}\right)+s\;\left(\text{time},28\right)+\mathrm{ns}\;\left(Z_{t}\right).$$

where *t* refers to the month of observation, and *Yt* refers to monthly cases of hospital admissions and mortality because of COPD, respectively. *Log*[*E* (*Yt*)] denotes the estimated monthly cases in month *t*, *X* refers to air pollutants (PM_10_, SO_2_ and NO_2_), *β* refers to the effect value, time refers to the unit of year and month, *Z*_*t*_ refers to meteorological data, and *s* denotes the cubic smoothing spline. Because the data were small, a *p*‐value of 0.05 was deemed significant.

This retrospective study followed the Strengthening the Reporting of Observational Studies in Epidemiology (STROBE) guidelines.^15^

## 3 RESULT

### 3.1 Characteristics of study subjects

During the 4‐year study period, 510 patients with COPD were eligible for inclusion. Of these, 95 COPD patients were dead during the study period.

The study included 368 male patients (72.16%) and 142 female patients (27.84%). The mean body mass index (BMI) was 22.68 ± 4.16 kg/m^2^ (range: 13.85–41.02 kg/m^2^). The proportion of the group with overweight was 25.89%. The proportion of the group with lower education (less than senior high school) was 69.22%. The proportion of the group with a high education (senior high school and above) was 30.78%. The mean number of smoking packs was 14 packs/year, and the mean age was 76.52 ± 7.98 year (range:44–90 year). The major complication among the COPD patients were hypertension and diabetes. The mean monthly number of COPD hospitalisations was 8.65 ± 3.26 (range:2–17). Among all cases, 95 patients died by 31 May 2010. The mean monthly COPD mortality was 1.9 ± 1.63 (range:0–9). The mean FEV_1_/FVC ratio was 65.24 ± 2.19%. The mean FEV_1_%pred was 61.12 ± 18.16%. The severity of COPD patients were graded according to the 2023 GOLD guidelines.^16^ The patients with moderate and severe COPD were predominant throughout the population. The mean FEV_1_ was 1.67 ± 0.32 L. The mean FVC was 2.85 ± 0.74 L. Further clinical characteristics of patients are reported in Tables 1 and 2.

**TABLE 1:** Summary of COPD patients in Beijing, China, 2006–2009.

COPD patients	*n* (%) (total *n* = 510)
Sex	
Male	368 (72.16%)
Female	142 (27.84%)
BMI	
≤18.49	78 (15.29%)
18.5–25	300 (58.82%)
>25	132 (25.89%)
Education	
Low education*	353 (69.22%)
High education*	157 (30.78%)
Smoking status	
Never smoker	130 (25.49%)
Ex‐smoker	267 (52.35%)
Current smoker	113 (22.16%)
Complication with hypertension	262 (51.37%)
Complication with diabetes	98 (19.22%)

*Abbreviations*: BMI, body mass index; COPD, chronic obstructive pulmonary disease.

Low education*: less than senior high school.

High education*: senior high school and above.

**TABLE 2:** The clinical characteristics of COPD patients in Beijing, China, 2006–2009.

COPD patients	n(%)	Mean	SD
Age (years)	510 (100%)	76.52	7.98
High (cm)	510 (100%)	165.43	8.15
Weight(kg)	510 (100%)	61.91	12.78
BMI	510 (100%)	22.68	4.16
COPD hospitalisations (case)	510 (100%)	8.65	3.26
COPD mortality (case)	95(18.63%)	1.92	1.63
FEV_1_/FVC %	498(97.65%)	65.24	2.19
FEV_1_%pred	498(97.65%)	61.12	18.16
GOLD 1	63(12.65%)	84.53	2.18
GOLD 2	275(55.22%)	57.39	8.29
GOLD 3	129(25.90%)	46.01	6.73
GOLD 4	31(6.23%)	18.65	4.19
FEV_1_(L)	498(97.65%)	1.67	0.32
FVC (L)	498(97.65%)	2.85	0.74

Abbreviations: BMI, body mass index; COPD, chronic obstructive pulmonary disease; FEV_1_, forced expiratory volume in 1 s; FVC, forced vital capacity; SD, standard deviation.

GOLD 1, FEV_1_%pred ≥ 80%; GOLD 2, 50 ≤ FEV_1_%pred < 79%; GOLD 3, 30 ≤ FEV_1_%pred < 49%; GOLD 4, FEV_1_%pred < 30%.

### 3.2 Exposure to pollutants

The monthly mean concentration of PM_10_ was 141.53 ± 41.20 μg/m^3^. The monthly mean concentration of SO_2_ and NO_2_ were 44.19 ± 39.09 μg/m^3^ and 54.67 ± 12.25 μg/m^3^, respectively. Yearly mean concentration of PM_10_ and NO_2_ were all over the China Grade II standard for ambient air quality (PM_10_ 70 μg/m^3^ and NO_2_ 40 μg/m^3^) (GB3095–2012). The SO_2_ was within the China Grade II standard for ambient air quality (60 μg/m^3^). The mean temperature was 13.63 ± 11.41 (−12–40°C). The mean pressure and relative humidity were 10123.26 ± 91.57 (9854–10 453 0.1 hPa) and 52 ± 15.81% (4–181%), respectively (Table 3).

**TABLE 3:** Summary of air pollutants and meteorological conditions in Beijing, China, 2006–2009.

Variables	Mean (monthly)	SD	Percent(100%)					IQR (μg/m^3^)
			Min	25%	50%	75%	Max	
Air pollutants								
SO_2_ (μg/m^3^)	44.19	39.09	6.79	13.28	26.61	61.86	156.50	48.58
NO_2_ (μg/m^3^)	54.67	12.25	30.13	43.32	56.02	64.11	77.30	20.79
PM_10_ (μg/m^3^)	141.53	41.20	70.52	110.68	137.27	166.57	297.07	55.89
Meteorological data								
Temp (°C)	13.63	11.41	−12.00	3.20	14.90	23.50	40.00	20.30
P (0.1 hPa)	10123.26	91.57	9854	10 039	10 117	10 203	10 453	164
RH (100%)	52	15.81	4	30	53	74	181	44

Abbreviations: COPD, chronic obstructive pulmonary disease; IOR, inter‐quartile range; NO_2,_ nitrogen dioxide; P, pressure; PM_10_, particulate matter with an aerodynamic profile<10 μm; RH, relative humidity; SD, standard deviation; SO_2,_ sulfur dioxide; Temp, temperature.

Table 4 shows the results of Spearman correlations between meteorological variables and air pollutants over the 4‐year study period. It showed there was significant correlation among the pollutants and meteorological variables. SO_2_ was correlated with NO_2_, PM_10_ and meteorological variables (*p* < 0.05). NO_2_ was correlated with SO_2_, PM_10_ and meteorological variables (*p* < 0.05). Relative humidity was also correlated with temperature and pressure(*p* < 0.05).

**TABLE 4:** Spearman rank correlations between different indicators of particulate air pollution: 2006–2009.

Variables	SO_2_	NO_2_	PM_10_	Temp	P	RH
SO_2_	1.000	0.678*	0.591*	−0.0086*	0.753*	−0.689*
NO_2_		1.000	0.610*	−0.0.704*	0.673*	−0.381*
PM_10_			1.000	−0.434*	0.260	–0.490*
Temp(°C)				1.000	–0.922	0.696*
Pre (0.1 hPa)					1.000	–0.521*
RH (100%)						1.000

Abbreviations: NO_2,_ nitrogen dioxide; P, pressure; PM_10_, particulate matter with an aerodynamic profile<10 μg/m^3^; RH, relative humidity; SO_2,_ sulfur dioxide; Temp, temperature.

* *p* < 0.05.

**FIGURE 1:**
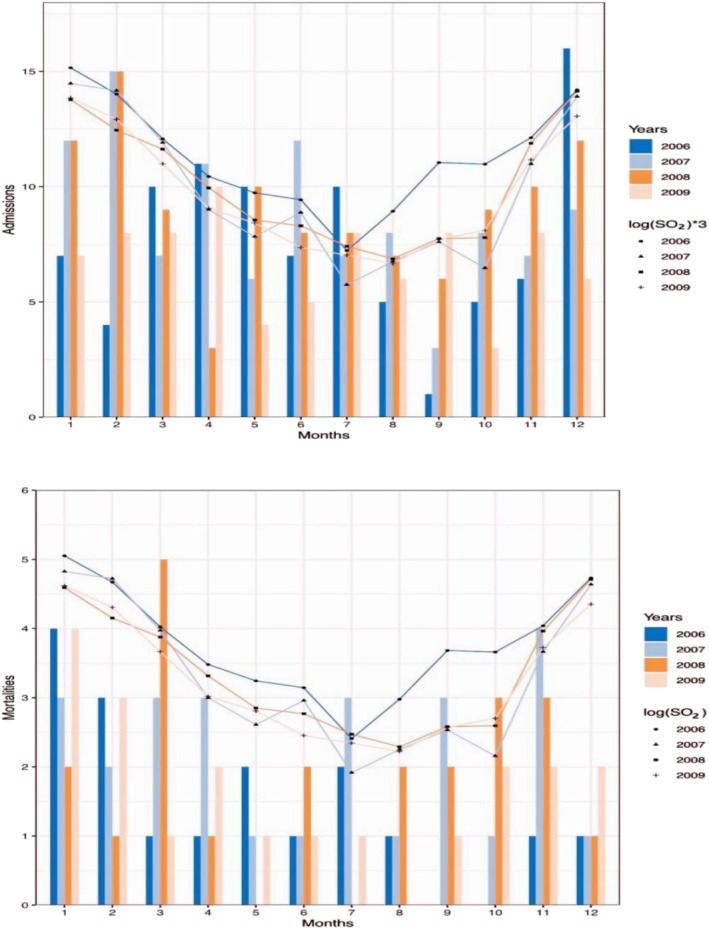
Time series of SO_2_ and COPD hospital admissions and mortality. SO_2_, sulfur dioxide; COPD, chronic obstructive pulmonary disease.

Figure 1 showed the time trends of air pollution and COPD hospital admissions and mortality. They all showed relatively stable seasonal trends annually, and they were higher in cold season and lower in warm season. Ever year from November to February of the next year, COPD hospital admissions, COPD mortality and the three air pollutants concentrations were all at the peak.

### 3.3 Effect on COPD hospital admissions

**FIGURE 2:**
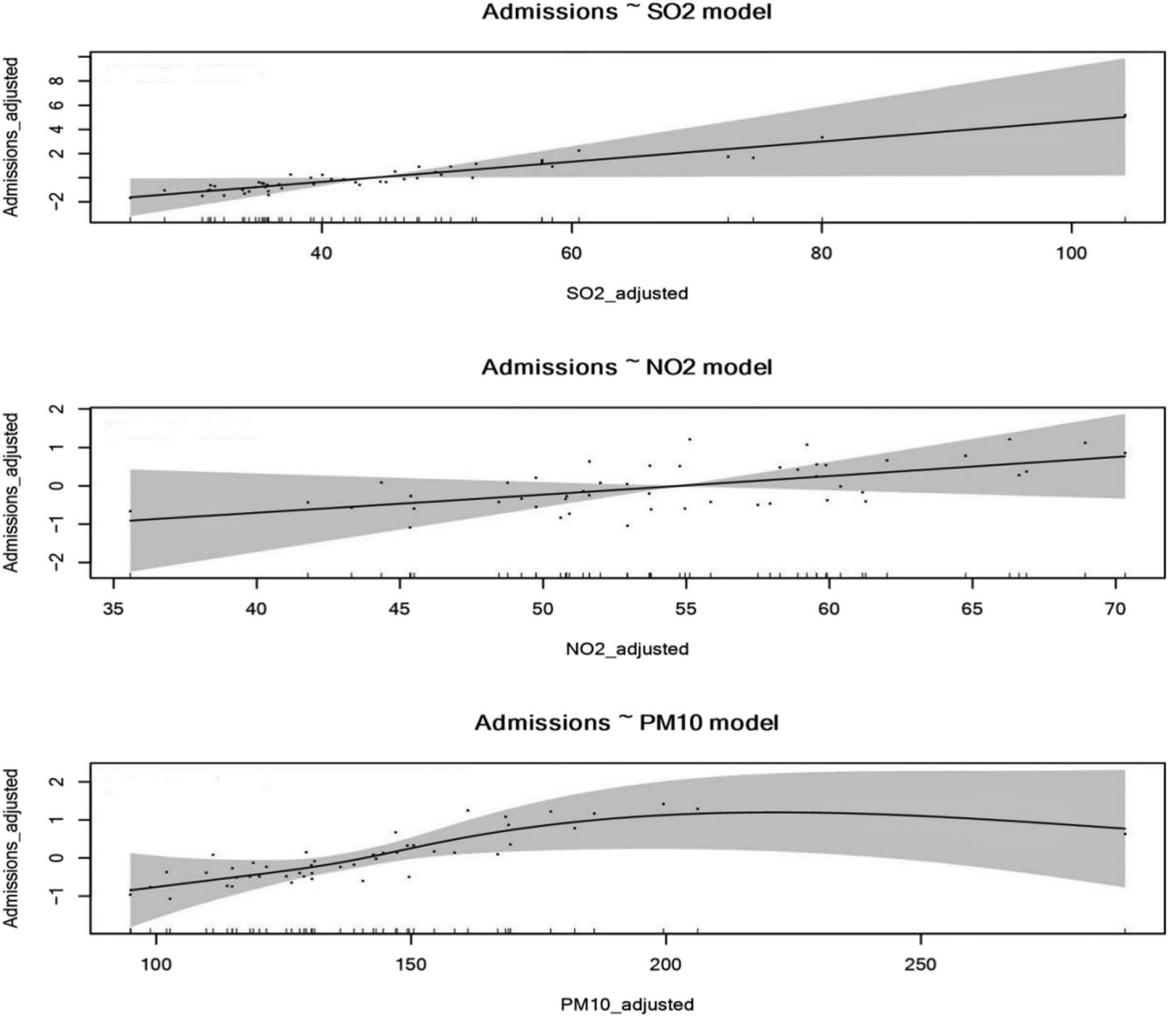
Air pollutants and COPD hospital admissions (single‐pollutant model). SO_2_, sulfur dioxide; NO_2_, nitrogen dioxide; PM_10_: particulate matter with an aerodynamic diameter ≤10 μm; COPD, chronic obstructive pulmonary disease.

We used the single‐pollutant model to assess the effects among SO_2,_ NO_2_ and PM_10_ with COPD hospital admissions. There were positive correlations among SO_2_, PM_10_ and COPD hospital admissions (SO_2_: *t* = 4.386, *p* = 0.023*; PM_10_: *t* = 7.817 *p* = 0.036*). An inter‐quartile range (IQR) changes of SO_2_ (48.58 μg/m^3^) and PM_10_ (55.89 μg/m^3^) were associated with an increase of 19.69% (7.14–25.16%) and 7.83% (3.72–10.34%) in hospital admissions for COPD, respectively (Table 5 and Figure 2).

**TABLE 5:** Correlation between particulate air pollution and COPD hospitalisations and mortality in Beijing, China, 2006–2009.

COPD effect	Pollutants	df	*β*(95% CI)	*t*	*P*‐value
**Single‐pollutant model**					
COPD hospital admissions	SO_2_	1.000	0.1969 (0.0714–0.2516)	4.386	0.023*
	NO_2_	1.045	‐‐‐	1.839	0.180
	PM_10_	2.558	0.0783 (0.0372–0.1034)	7.817	0.036*
COPD mortality	SO_2_	2.995	‐‐‐	4.426	0.228
	NO_2_	1.164	‐‐‐	2.420	0.249
	PM_10_	2.933	‐‐‐	4.069	0.297
**Multiple‐pollutant model**					
COPD hospital admissions (two air pollutants)	SO_2_	1.000	0.0931 (0.0543–0.2082)	3.926	0.045*
	NO_2_	1.453	‐‐‐	1.935	0.345
	SO_2_	1.000	‐‐‐	0.775	0.379
	PM_10_	2.540	‐‐‐	3.575	0.308
	NO_2_	1.000	‐‐‐	0.800	0.371
	PM_10_	2.686	‐‐‐	6.213	0.109
ALL air pollutants	SO_2_	1.000	‐‐‐	0.094	0.759
	NO_2_	1.000	‐‐‐	0.103	0.749
	PM_10_	2.568	‐‐‐	3.606	0.305
COPD mortality (two air pollutants)	SO_2_	1.573	‐‐‐	1.170	0.416
	NO_2_	1.000	‐‐‐	1.271	0.260
	SO_2_	1.000	‐‐‐	0.044	0.834
	PM_10_	2.940	‐‐‐	3.930	0.331
	NO_2_	1.000	‐‐‐	0.454	0.500
	PM_10_	2.655	‐‐‐	2.362	0.529
ALL air pollutants	SO_2_	1.000	‐‐‐	0.933	0.334
	NO_2_	1.000	‐‐‐	1.150	0.284
	PM_10_	2.608	‐‐‐	2.711	0.445

*Note*: Adjust for mean temperature, pressure and relative humidity.

Abbreviations: *β*, effect estimate; CI, confidence interval; df, degrees of freedom; NO_2,_ nitrogen dioxide; PM_10_, particulate matter with an aerodynamic profile<10 μg/m^3^; SO_2,_ sulfur dioxide.

* *p* < 0.05.

**FIGURE 3:**
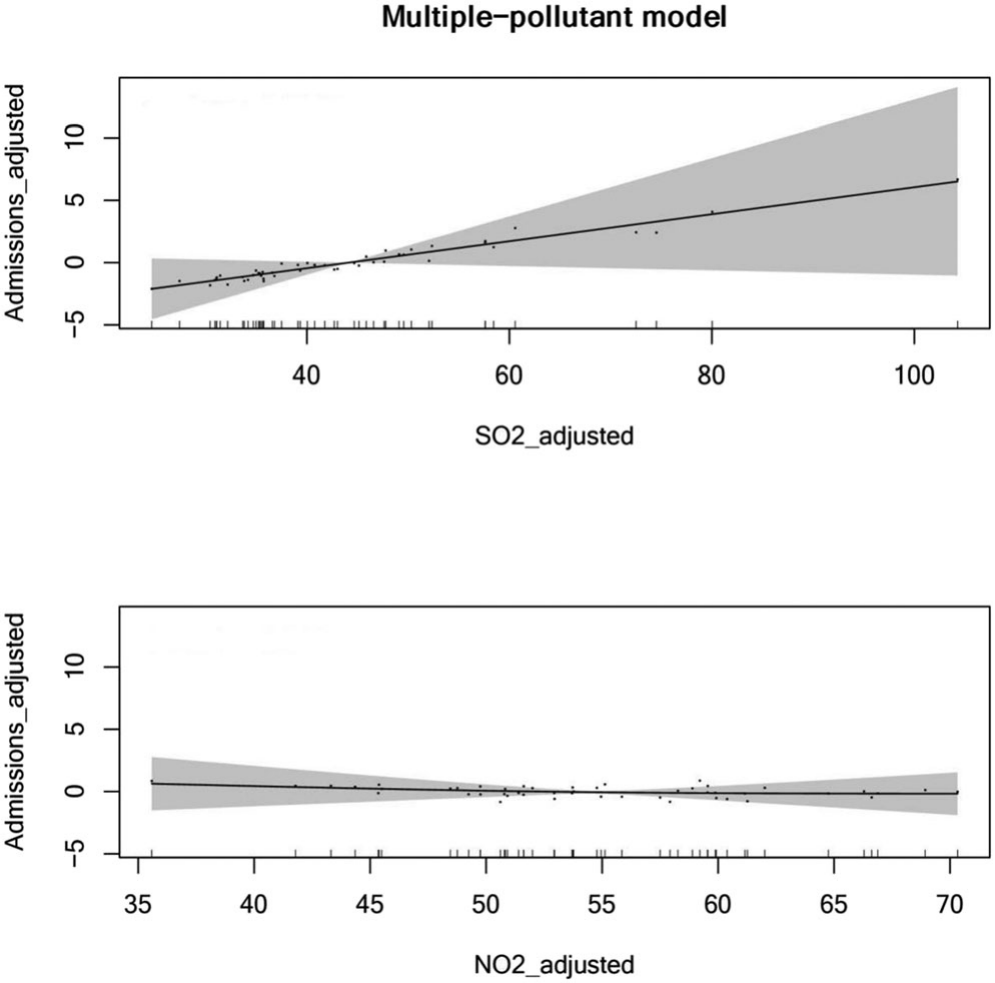
Air pollutants and COPD hospital admissions (multiple‐pollutant model: SO_2_ and NO_2_). SO_2_, sulfur dioxide; NO_2_, nitrogen dioxide; COPD, chronic obstructive pulmonary disease.

In the multiple‐pollutant models, COPD hospital admissions was only positively associated with SO_2_ and NO_2_ combinations (SO_2_: *t* = 3.926, *p* = 0.045*; NO_2_: *t* = 1.935, *p* = 0.345). An IQR change of SO_2_ (48.58 μg/m^3^) was associated with an increase of 9.31% (5.43–20.82%) in hospital admissions because of COPD (Table 5 and Figure 3). There was no correlation between three pollutant combinations and COPD hospital admissions (Table 5).

### 3.4 Effect on COPD mortality

**FIGURE 4:**
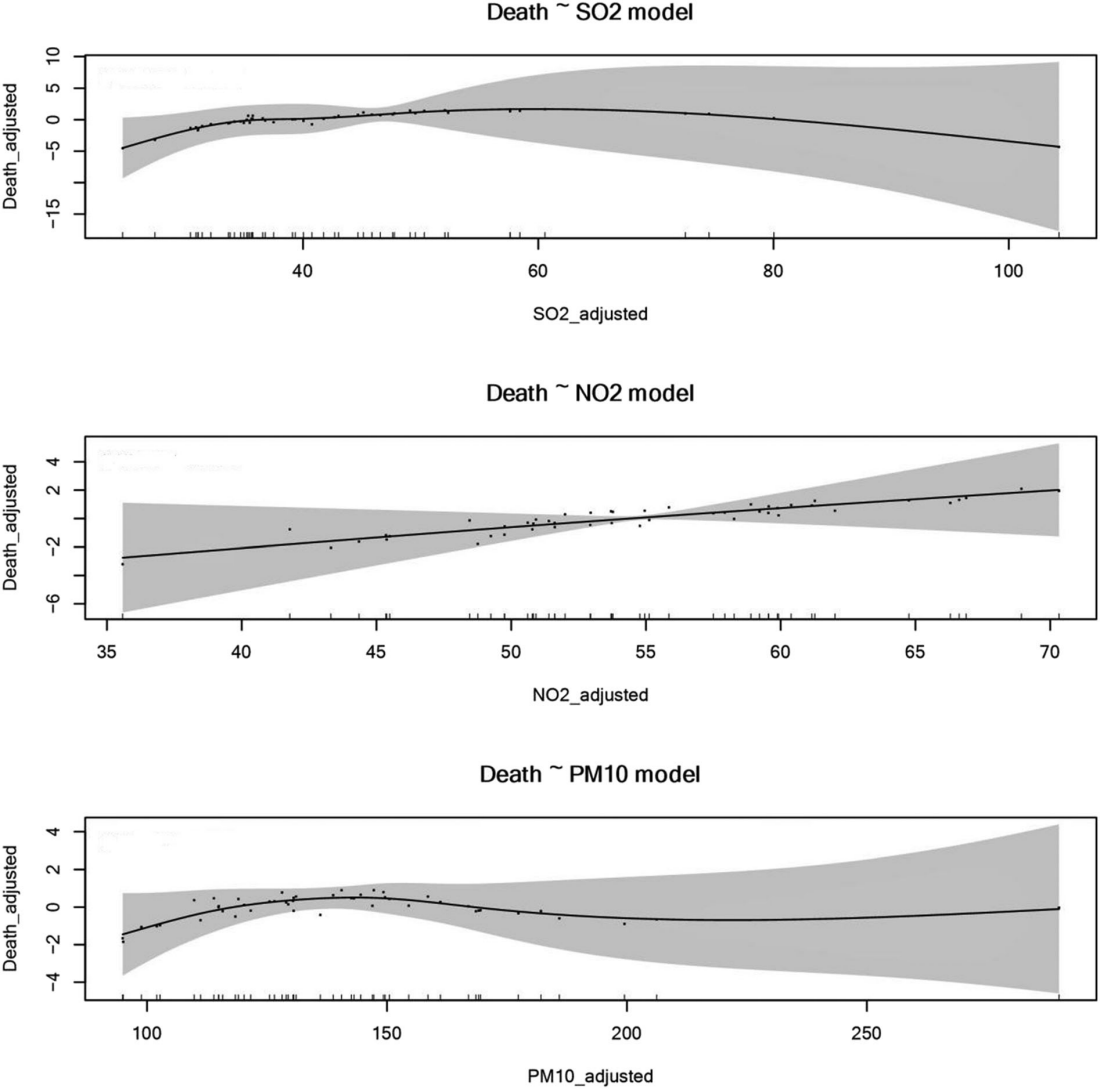
Air pollutants and COPD mortality (single‐pollutant model). SO_2_, sulfur dioxide; NO_2_, nitrogen dioxide; PM_10_: particulate matter with an aerodynamic diameter ≤10 μm; COPD, chronic obstructive pulmonary disease.

We used the single‐pollutant model to assess the effects among SO_2,_ NO_2_ and PM_10_ with COPD mortality. There was no correlation between air pollution and COPD mortality in the single‐pollutant model (Table 5 and Figure 4).

In the multiple‐pollutant models, there was no correlation between two pollutant combinations, three pollutant combinations and COPD mortality (Table 5).

The single and multiple models were all adjusted for the mean temperature, pressure and relative humidity.

## 4 DISCUSSION

In this study, we present one of the few reports regarding the effects of air pollution on hospital admissions and mortality because of COPD in Beijing, China. We found that over the 4‐year study period, COPD hospital admissions and mortality showed relatively stable seasonal trends annually. They were higher in cold season and lower in warm season. In recent decades, air pollution has cause considerable public concern in China, especially in Beijing. Yearly concentrations of NO_2_ and PM_10_ exceeded the Chinese ambient air quality standards and were far higher than those reported in the USA and Europe. Yearly concentrations of SO_2_ were higher than those reported in the USA and Europe, too.^17^ Luo et al.^18^ found that PM_10_ pollution occurred mainly in Spring and Winter. Cichowicz et al.^19^ reported that PM_10_, SO_2_, NO_2_ and CO were higher in winter. And the higher levels of pollution in winter month are presented by PM_10_ concentration that exceed the limit values. This study indicated that PM_10_ and NO_2_ were higher than SO_2_. The reason may be the source of PM_10_ and NO_2_ is from motor vehicle emissions, coal burning and frequent dust storms, while combustion of sulfur‐contain fuels and production of waste gas in chemical industry dominated SO_2_ emission. Beijing has a heavy industrial structure and massive motor traffic, resulting in large quantities of industrial emissions for PM_10_ and NO_2_. Air pollution is worse in winter because of coal burning for heating in Beijing. Pollutants are not easy to spread because winter is dominated by downdraft, and temperature inversion is easy to occur. Fortunately, people paid increasing attention to the effects of air pollution, and we observe changes of air pollution in Beijing through the efforts of the government. Therefore, we aimed to update the association between air pollution and COPD hospital admissions and mortality and compared the effect after 10 years in Beijing, China. Unfortunately, the data of 10 years late have not been fully collected.

This study showed that an increase of 10 μg/m^3^ in SO_2_ and PM_10_ was associated with an increase of 4.053% (95% CI: 1.470–5.179%) and 1.401% (95%CI: 0.6656–1.850%) in COPD hospital admissions, respectively. In the multiple‐pollutant model (SO_2_ and NO_2_ combinations), there was only a positive correlation between SO_2_ and COPD hospital admissions. An increase of 10 μg/m^3^ in SO_2_ was associated with an increase of 1.916% (95% CI 1.118%–4.286%) in COPD hospital admissions. This findings were similar to the previous studies. Dabrowiecki P et al.^20^ reported that during 21 days after exposure, an increase of 10 μg/m^3^ in SO_2_ and PM_10_ was associated with an increase of 14.5% (95% CI: 3.8–26.2%) and 2.8% (95%CI: 0.8–4.9%) in COPD hospital admissions, respectively. Ghanbari et al.^21^ reported that during 1 year exposure, an increase of 10 μg/m^3^ in SO_2_ was associated with an increase of 0.5% (95% CI: 0%–1%) in COPD hospital admissions. Studies indicated that short‐term exposure to air pollution may also indicate risk of COPD hospital admissions. Li et al.^17^ reported that each 10 μg/m^3^ increase in SO_2_ and PM_10_ concentrations corresponded to an increase in COPD hospitalisation of 2.07%(95% CI: 1.00–3.15%) at lag 0–1 days and 0.92%(95% CI: 0.55–1.30%) at lag 0–7 days, respectively in Beijing, China. Mercan et al. reported that a 10 μg/m^3^ increase in the current day (lag 0) concentrations of SO_2_ and PM_10_ corresponded to an increase of 6.5% (95% CI: 5.6–7.5%) and 2.9% (95% CI: 2.2–3.5%) for COPD hospitalisations in Anatolia, Turkey.^22^ A meta‐analysis concluded that a 10 ug/m^3^ increase in SO_2_ was associated with a 2.1% (95%CI: 0.7–3.5%) increase in COPD hospital admission.^23^

This study did not found correlation between NO_2_ and COPD hospital admissions. However, many studies conducted NO_2_ and COPD hospital admissions. Li et al.^17^ reported that each 10 μg/m^3^ increase in NO_2_ concentrations corresponded to an increase in COPD hospitalisation of 3.03% (95% CI: 1.82–4.26%) at lag 0–6 days. In Iran, Hanieh et al. found that a 10 μg/m^3^ increase in NO_2_ concentrations corresponded to an increase of 4.9% (95% CI: 1.0–9.0%) in COPD hospital admissions.^24^ We also found no association between the air pollutants investigated and COPD mortality. Yan et al. reported a significant positive association of SO_2,_ NO_2_ and PM_10_ with deaths because of COPD. In their single pollutant model, each 10 μg/m^3^ increase in SO_2,_ NO_2_ and PM_10_ levels increased COPD mortality by 4.299%(95%CI:0.978–7.729%), 1.816%(95%CI: 0.515–3.313%) and 0.583%(95%CI: 0.055–1.113%) at lag0–3, respectively.^25^ Chen et al. had reported that an increase in SO_2_ (8 μg/m^3^) and NO_2_ (8 μg/m^3^) was associated with an increase of 4.3% (95%CI: 2.1–6.4%) and 3.6% (95%CI: 1.7–5.6%) in COPD mortality in patients aged 60 years and older.^26^ Meng et al.^27^ reported that an increase of 10 μg/m^3^ in SO_2,_ NO_2_ and PM_10_ was associated with an increase in COPD mortality of 1.3%(95%CI:0.61–1.99%), 1.78%(95%CI: 1.1–2.46%) and 0.78% (95%CI: 0.13–1.42%), respectively, in four cities in China. Moolgavkar et al. found a significant positive association between PM_10_ and the risk of COPD related death with an increase in mortality of 2.66% (95%CI: 0.12–0.52%) per 25 μg/m^3^ increase in PM_10._ ^28^ However, Bateson et al. did not find association between PM_10_ and COPD mortality in the United States.^29^ The conflicting findings in comparison with our study might be because of the different chemical composition of air pollutants in different regions, which may result in different effects of COPD hospitalisations and mortality.

In this study, the majority of the summary estimates for COPD hospital admissions were in the order of SO_2_ > PM_10_ > NO_2._ The yearly mean concentration of SO_2_ was low compared with the other two pollutants, but SO_2_ had a stronger effect on COPD hospital admissions. The reason for this phenomenon remains unclear. Regional differences may contribute to these results. In our study, the small sample size and single study area may be the reason for our results. SO_2_ was mainly came from burning of fossil fuels. It is very soluble and can be absorbed more when people move. The patients with COPD often have difficulty in breathing; they are more sensitive and suffer more damage. It can exert immediate effects on the respiratory tract at early contact. Particulate matter exposures may cause production of reactive oxygen and inflammatory factors in alveolar macrophages in humans. NO_2_ exposures can exacerbate existing respiratory disease by impairing the functions of epithelial cells and alveolar macrophages, contributing to airway inflammation.^30^, ^31^ The specific mechanism of air pollution and COPD is unclear. It is suggested that the exacerbation of inflammation, oxidative stress and immunosuppression may play important roles in COPD.^32^, ^33^, ^34^ Further studies are needed to demonstrate the mechanism of air pollution and COPD.

Our study had several limitations. First, this study was conducted in one region of China and in one hospital, and included a small number of patients with COPD. Second, other important unknown and unmeasured factors may have affected the results. For example, the data on body mass index (BMI), smoking, socioeconomic status and education level collected in this study may play an important role as effect modifiers. Finally, personal exposure to outdoor and indoor air pollution was inconsistent among the selected COPD patients. The last but not the least, personal exposure to outdoor air pollution and indoor air pollution may not accurately represented exposure levels in general population.

Fortunately, people in China have begun consideration to the health effects of air pollution and COPD, especially PM_2.5_ (particulates with an aerodynamic diameter of ≤2.5um). Many studies have suggested that the adverse effects on COPD because of to PM_2.5_ are greater than those of PM_10._ ^35^, ^36^, ^37^ In 2020, our team had conducted a meta‐analysis on the association between PM_2.5_ and COPD hospitalisations and mortality.^10^ Additionally, our findings regarding the effect of PM_2.5_ on lung function in COPD are currently being summarised. Elucidating the mechanism of the effects of air pollution in COPD will be the main direction of our future research. Air pollution control is also a topic priority in the future.

## 5 CONCLUSION

This study supported the association between air pollution and COPD hospital admissions. SO_2_ and PM_10_ may be important factors for the increase in COPD hospital admissions in Beijing, China. Air pollution is high, and it emphasises the need to reduce air pollution in Beijing, China. Further detailed studies are needed to confirm the present findings and to clarify the mechanisms.

## AUTHOR CONTRIBUTION

Rui‐xia, Zhu conducted literature collection and selection. Rui‐xia Zhu wrote the manuscript. Jin Chen provided valuable advice on the entire article.

## CONFLICT OF INTEREST STATEMENT

The authors declare that there is no conflict of interest.

## FUNDING

This research received no specific grants from any funding agency in the public, commercial, or not‐for‐profit sectors.

## ETHICS STATEMENT

This was a retrospective study; we waived the requirement for ethical approval and informed patient consent from the Ethics Committee of Peking University Third Hospital.

## Data Availability

All data generated or analysed during this study are included in this article. Further demand can be directed to the corresponding author in email.
